# Unravelling the nutritional and health benefits of marketable winged termites (*Macrotermes* spp.) as sustainable food sources in Africa

**DOI:** 10.1038/s41598-024-60729-9

**Published:** 2024-05-01

**Authors:** Xavier Cheseto, Brian O. Ochieng, Sevgan Subramanian, Chrysantus M. Tanga

**Affiliations:** International Centre of Insect Physiology and Ecology (Icipe), P.O. BOX 30772-00100, Nairobi, Kenya

**Keywords:** Winged termites, Marketable products, Flavonoids, Nutrient-rich profile, Malnutrition, Food security, Chemical biology, Chemistry

## Abstract

Termites are widely distributed globally and serve as a valuable food source in many countries. However, information on the myriad nutritional benefits of processed termite products in African markets remain largely unexploited. This study evaluated the phytochemicals, fatty acids, amino acids, minerals, vitamins and proximate composition of the edible winged termites (*Macrotermes* spp.) from three major Counties of Kenya. A total of 9 flavonoids, 5 alkaloids, and 1 cytokinin were identified. The oil content varied from 33 to 46%, exhibiting significant levels of beneficial omega 3 fatty acids, such as methyl (9*Z*,12*Z*,15*Z*)-octadecatrienoate and methyl (5*Z*,8*Z*,11*Z*,14*Z*,17*Z*)-eicosapentaenoate, ranging from 82.7–95.1 to 6.3–8.1 µg/g, respectively, across the different regions. Four essential and cereal-limiting amino acids lysine (1.0–1.3 mg/g), methionine (0.08–0.1 mg/g), leucine (0.6–0.9 mg/g) and threonine (0.1–0.2 mg/g), were predominant. Moreover, termites had a rich profile of essential minerals, including iron (70.7–111.8 mg/100 g), zinc (4.4–16.2 mg/100 g) and calcium (33.1–53.0 mg/100 g), as well as vitamins A (2.4–6.4 mg/kg), C (0.6–1.9 mg/kg) and B12 (10.7–17.1 mg/kg). The crude protein (32.2–44.8%) and fat (41.2–49.1%) contents of termites from the various Counties was notably high. These findings demonstrated the promising nutrients potential of winged termites and advocate for their sustainable utilization in contemporary efficacious functional food applications to combat malnutrition.

## Introduction

Many developing countries and emerging economies are facing significant challenges related to food insecurity and a growing prevalence of diet-related chronic diseases^1,2^. These challenges are driven by factors such as rising populations, high food prices, overreliance on few protein sources, climate change, unequal trade, food waste, and unstable food supplies^3^. These issues are expected to become even more pressing in the future, leading to increased food shortages and diseases. Despite the fact that malnutrition is the leading cause of disease worldwide, Kenya has a high prevalence of chronic and acute malnutrition, with 26% and 5% of children under the age of five affected, respectively^4^. In light of these challenges, scientists, policy makers, and the United Nations have emphasized the need for dietary diversification. Edible insects, such as termites, have been proposed as a potential solution to these issues due to their ability to alleviate undernutrition in developing nations and ensure future food security^5^ and their smaller ecological footprints^6^. This has prompted research into the nutritional and health benefits of several edible insects and calls for further exploration.

Edible insects have been entangled in human dietary traditional heritage from the antique times^7^ and plays a pertinent role as nutritious diets among many communities worldwide^8^. They are not only consumed during times of scarcity, but also regularly as part of traditional diets^9^. African cultures have adopted insect consumption because of their taste, cultural activities, nutritive value, food supplement when staple food is limited^10^, and for entomotherapeutic and medicinal values^11^. Their anecdotal health and nutritional benefits have since been explored and linked to a myriad of bioactive, antioxidative properties from their chitinous envelop^12^ and dense nutritional profiles featuring high proteins, amino acids, healthy fatty acids, vitamins, phytosterols and essential minerals^13^.

Termites are considered a customary delicacy in Africa including cultures of the western Kenya. A typical colony comprises nymphs, workers, queen, and soldiers but the most commonly consumed form is the mature winged alates^14^. In Kenya, several edible termite species have been reported such as *Macrotermes nigeriensis*, *Pseudacanthotermes militaris* (Hagen), *Macrotermes bellicosus* (Smeathman), *Macrotermes subhylanus* (Rambur), *Pseudacanthotermes spiniger* (Sjostedt) and the others such as *Neotermes* spp., *Coptotermes* spp., and *Cryptotermes* spp. They plentifully emerge during the first rains marking the end of the dry periods in the early mornings, evenings or upon attraction to light at night, during which residents avidly collect them for food^15,16^. Traditionally, the residents capitalized on the nocturnal nature of the termites by setting bowls of water below light sources as traps^17^. Moreover, the villagers have mastered the art of inducing the emergence of these insects even during the dry seasons to satisfy the outstanding market demand and sustain the established enterprise^15^. Commonly embraced processing techniques for termites marketed in many communities include de-winging, blanching, sun-drying, roasting, frying, toasting and salting, or grinding into powder^9,17^.

The nutritional quality, protein digestibility and mineral bioavailability of termites have been well documented^9,15^. In many countries, consumption of termites is driven by their health and nutritional benefits, which make them highly recommendable for breastfeeding mothers and children under the age of 5 years due to richness in protein and iron^9^. Thus, they are considered as excellent candidates for alleviating malnutrition and micronutrient deficiency in many developing countries^9^. Previously, antimicrobial peptides have been isolated from *Pseudocanthotermes spiniger*, *Reticulitermes* spp. and *Odontotermes formosanus* Shiraki^18^. Similarly, terpenes have been extracted from *Nasutitermes* spp. and *Prorhinotermes* spp., while alcohol extracts of *Macrotermes estherae* Desneux and *Microtermes obes* Holmgren have been shown to express antimicrobial activities^18^. Despite their established use in traditional medicine for treating myriad of medical conditions, a comprehensive understanding of termites’ bioactive components remain elusive^11^. Additionally, the chemical composition of termites exhibits substantial inter- and intra-species variations, influenced by factors such as species identity, environmental conditions, geographical location, feeding substrate, and the developmental life stages of the insects^16^. In light of this knowledge gap, this study aimed to investigate, document the diverse phytochemicals, dietary value and potential health benefits associated with winged termites (*Macrotermes* spp.) in three Counties of Western Kenya. Elucidating this information can contribute to promoting informed consumer awareness and demystifying the long-standing yet poorly understood nutritional and potentially therapeutic properties that have underpinned human utilization of termites throughout history.

## Results

### Phytochemical contents

A total of 9 flavonoids, 5 alkaloids and 1 cytokinin were identified from the termites obtained from the three Counties (Table 1). Significant variations (*p* < 0.05) in the flavonoid contents (except luteolin), alkaloids and cytokinin were evident from the termites sourced from the different locations. The levels of flavonoids; rutin, quercetin, kaempferol, 4-hydroxybenzoic acid, naringenin, alkaloids; solanine, tomatidine, solanidine, solasodine and cytokinin; zeatin were significantly higher in samples collected in Kakamega County compared to the other two Counties.

**Table 1:** Phytochemical composition (µg/g DM) of termites from three Counties in Kenya.

Compounds	Bungoma	Kakamega	Homa Bay	F_(2,6)_	p-value
Flavonoids					
Rutin	2.1 ± 0.02^b^	2.4 ± 0.02^c^	1.5 ± 0.03^a^	283.3	0.001
Quercetin	0.9 ± 0.01^b^	1.0 ± 0.02^c^	0.6 ± 0.02^a^	87.6	0.001
Kaempferol	1.2 ± 0.01^b^	1.4 ± 0.04^c^	0.8 ± 0.03^a^	99.7	0.001
Luteolin	0.4 ± 0.03^a^	0.4 ± 0.01^a^	0.4 ± 0.02^a^	1.7	ns
4-Hydroxybenzoic acid	2.4 ± 0.01^b^	2.6 ± 0.03^b^	1.8 ± 0.01^a^	64	0.001
Caffeic acid	0.7 ± 0.01^b^	0.1 ± 0.00^a^	0.1 ± 0.01^a^	1989.2	0.001
Ferulic acid	0.8 ± 0.02^b^	0.3 ± 0.02^a^	0.7 ± 0.04^b^	94.7	0.001
Naringenin	0.2 ± 0.00^ab^	0.2 ± 0.00^b^	0.2 ± 0.01^a^	8.9	0.05
Apigenin	1.9 ± 0.02^b^	0.8 ± 0.01^a^	0.7 ± 0.04^a^	663	0.001
Alkaloids					
Solanine	2.2 ± 0.01^b^	2.7 ± 0.02^c^	2.0 ± 0.05^a^	118.5	0.001
Tomatidine	9.6 ± 0.17^b^	10.9 ± 0.22^c^	7.5 ± 0.38^a^	40.5	0.001
Solanidine	4.1 ± 0.05^b^	4.9 ± 0.22^c^	3.3 ± 0.18^a^	23.9	0.01
Solasodine	9.4 ± 0.10^b^	10.4 ± 0.10^b^	8.3 ± 0.40^a^	18.8	0.01
Chaconine	2.2 ± 0.01^c^	2.6 ± 0.02^b^	1.8 ± 0.06^a^	116.7	0.001
Cytokinin					
Zeatin	2.3 ± 0.01^b^	2.5 ± 0.01^c^	1.8 ± 0.06^a^	97	0.001

*DM* dry matter.

Mean values with different subscripts along each row differ significantly at p > 0.05.

### Oil yields from winged termites from three different localities

**Figure 1:**
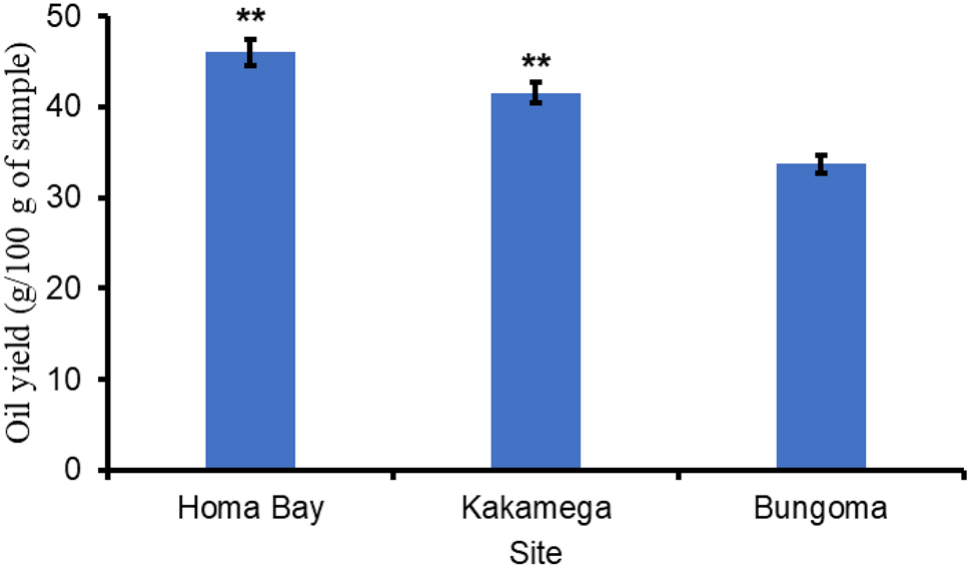
Termites oil yield in the three study sites. Homa Bay, Kakamega and Bungoma Counties. Error bars indicate the standard error of the mean. Asterisks indicate significant difference between the means: **p < 0.01.

Termites sourced from Homa Bay County had 46% oil yield compared to 42% and 33% for Kakamega and Bungoma Counties. There was no significant difference in termite oil yields between Homa Bay and Kakamega Counties samples, though both were significantly higher compared to samples from Bungoma County (*F*_2,6_ = 17.8, *p* = 0.003) (Fig. 1).

### Fatty acid composition

**Figure 2:**
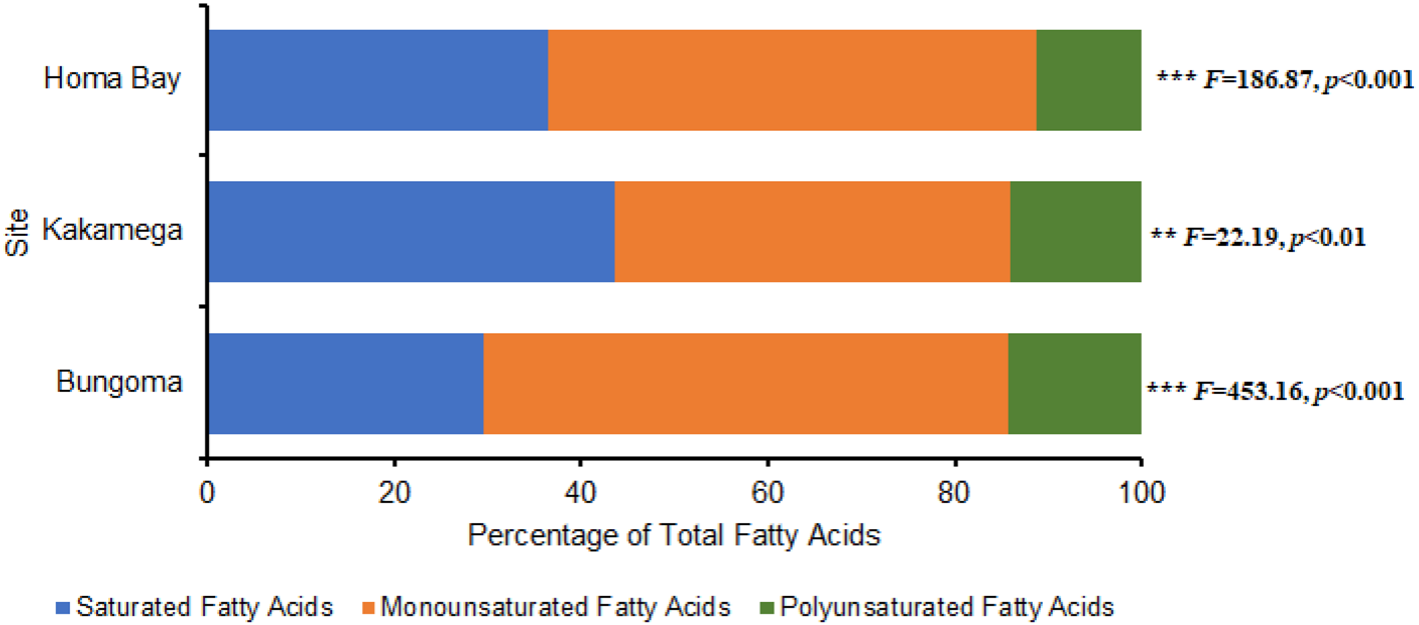
Fatty acid methyl esters (FAMEs) composition in termite’s oils from three different localities (Homa Bay, Bungoma and Kakamega). ***Denotes significantly different at 0.001.

The fatty acid analysis of winged termite oils showed similar amounts of saturated fatty acids (SFAs), monounsaturated fatty acids (MUFAs), and polyunsaturated fatty acids (PUFAs). The major constituents in each category were SFA methyl hexadecanoate (51–73%), MUFA methyl (9*Z*)-octadecenoate (47–64%), and PUFA methyl (9*Z*,12*Z*)-octadecadienoate (72–80%) as indicated by Fig. 2 and Table 2.

**Table 2:** Fatty acid composition of termite oils (µg/g) analyzed by gas chromatography coupled to mass spectrometry (GC–MS).

RT	Compound name	ω-n (∆n)	Bungoma	Kakamega_	Homa Bay	*F*_(2,6)_	*p*-value
17.79	Methyl 2,6-dimethyltridecanoate	Iso-dimethyl-C13:0	0.5 ± 0.02^a^	1.2 ± 0.04^a^	2.3 ± 0.22^b^	31.952	0.001
19.24	Methyl dodecanoate	C12:0	3.4 ± 0.08^a^	36.5 ± 0.88^c^	21 ± 1.94^b^	120.94	0.001
20.39	Methyl tridecanoate	C13:0	0.9 ± 0.06^a^	1.0 ± 0.07^a^	0.8 ± 0.03^a^	2.3151	ns
21.06	Methyl 12-methyltridecanoate	Iso-methyl-C13:0	0.7 ± 0.03^a^	1.9 ± 0.06^b^	1.5 ± 0.20^b^	16.232	0.01
21.48	Methyl tetradecanoate	C14:0	159.6 ± 19.59^a^	179.7 ± 22.05^a^	133.6 ± 10.23^a^	0.9534	ns
22.20	Methyl 13-methyltetradecanoate	Iso-methyl-C14:0	12.3 ± 0.77^a^	28.4 ± 1.78^b^	21.4 ± 2.53^a,b^	12.856	0.01
22.57	Methyl pentadecanoate	C15:0	24.9 ± 2.32^a^	30.7 ± 2.86^a^	24.2 ± 2.10^a^	1.3984	ns
22.94	Methyl 3-methylpentadecanoate	Iso-methyl-C15:0	0.1 ± 0.02^a^	0.3 ± 0.04^b^	0.1 ± 0.01^a^	17.547	0.01
23.70	Methyl hexadecanoate	C16:0	827.7 ± 77.14^a^	974.4 ± 23.65^a^	1205.7 ± 37.54^b^	5.2794	0.05
24.57	Methyl heptadecanoate	C17:0	32.3 ± 4.14^a^	34.0 ± 5.63^a^	37.1 ± 3.08^a^	0.0615	ns
25.73	Methyl octadecanoate	C18:0	3.8 ± 0.45^a^	509.4 ± 16.23^b^	479.9 ± 18.23^b^	23.712	0.01
26.41	Methyl nonadecanoate	C19:0	19.9 ± 0.65^a^	23.7 ± 0.78^a^	21.5 ± 2.75^a^	0.836	ns
27.26	Methyl 18-methylnonadecanoate	Iso-methyl-C19:0	98.1 ± 7.86^a^	129.2 ± 19.23^a^	102.2 ± 9.53^a^	0.6619	ns
28.07	Methyl heneicosanoate	C21:0	10.5 ± 0.55^a^	18.4 ± 0.97^b^	19.9 ± 1.84^a,b^	11.108	0.01
28.87	Methyl docosanoate	C22:0	11.4 ± 0.95^a^	17.7 ± 1.47^b^	17.1 ± 0.81^a,b^	6.6001	0.05
29.63	Methyl tricosanoate	C23:0	8.6 ± 0.80^a^	13.8 ± 1.28^a^	10.0 ± 1.55^a^	3.0762	ns
30.45	Methyl tetracosanoate	C24:0	10.9 ± 1.00^a^	17.4 ± 1.61^b^	13.2 ± 0.83^a,b^	5.1527	0.05
32.48	Methyl hexacosanoate	C26:0	2.1 ± 0.25^a^	4.4 ± 0.52^a^	5.1 ± 0.79^a^	5.0118	ns
	∑SFA		1227.7 ± 61.44	2022.1 ± 380.01	2116.6 ± 94.16		
21.20	Methyl (5*Z*)-dodecenoate	C12:1 (n-7)	3.1 ± 0.04^a,b^	4.3 ± 0.55^b^	1.8 ± 0.21^a^	6.2901	0.05
21.25	Methyl (9*Z*)-tetradecenoate	C12:1 (n-5)	8.1 ± 0.60^a^	8.9 ± 0.02^a^	9.9 ± 0.83^a^	1.1294	ns
23.44	Methyl (9*Z*)-hexadecenoate	C16:1 (n-7)	472.9 ± 29.55^a^	508.3 ± 31.76^a^	566.8 ± 30.78^a^	1.3008	ns
24.16	Methyl (6*Z*)-7-methylhexadecenoate	Iso-methyl-C16:1(n-10)	0.9 ± 0.08^a^	22.0 ± 2.03^b^	22.8 ± 3.91^b^	15.963	0.01
24.37	Methyl (9*Z*)-heptadecenoate	C17:1 (n-8)	514.4 ± 16.90^b^	36.8 ± 1.21^a^	31.5 ± 1.65^a^	530.49	0.001
25.40	Methyl (9*Z*)-octadecenoate	C18:1 (n-9)	1276.6 ± 56.07^a^	1335 ± 24.63^a^	1478.8 ± 56.23^a^	0.5148	ns
25.59	Methyl (9*E*)-octadecenoate	C18:1 (n-9)	1.0 ± 0.12^a^	1.2 ± 0.14^a^	871 ± 20.04^a^	78.568	0.001
26.21	Methyl (10*Z*)-nonadecenoate	C19:1 (n-9)	26.2 ± 2.18^a^	23.6 ± 1.96^a^	23.7 ± 0.78^a^	0.4517	ns
27.05	Methyl (11*Z*)-eicosenoate	C20:1 (n-9)	21.9 ± 0.68^a^	27.8 ± 0.87^a^	39.4 ± 2.47^b^	21.821	0.01
	∑MUFA		2324.9 ± 140.85	1967.9 ± 131.58	3045.8 ± 136.04		
23.27	Methyl (7*Z*,10*Z*)-hexadecadienoate	C16:2 (n-6)	2.7 ± 0.42^a^	4.0 ± 0.63^a^	3.1 ± 0.26^a^	1.4989	ns
25.32	Methyl (9*Z*,12*Z*)-octadecadienoate	C18:2(n-6)	453.5 ± 37.69^a^	474.3 ± 39.41^a^	525.4 ± 48.34^a^	0.5148	ns
25.80	Methyl (9*Z*,12*Z*,15*Z*)-octadecatrienoate	C18:3 (n-3)	92.1 ± 10.86^a^	82.7 ± 9.75^a^	95.1 ± 2.28^a^	0.3893	ns
25.83	Methyl (9*Z*,11*E*,13*E*)-octadecatrienoate	C18:3 (n-5)	28.5 ± 2.47^a^	77.1 ± 6.66^b^	11.5 ± 0.71^a^	45.441	0.001
26.74	Methyl (5*Z*,8*Z*,11*Z*,14*Z*)-eicosatetraenoate	C20:4 (n-6)	8.2 ± 0.68^a^	11.5 ± 0.96^a^	12.8 ± 0.96^a^	4.9752	ns
26.80	Methyl (5*Z*,8*Z*,11*Z*,14*Z*,17*Z*)-eicosapentaenoate	C20:5 (n-3)	6.3 ± 0.45^a^	8.1 ± 0.57^a^	6.7 ± 0.83^a^	1.4416	ns
	∑PUFA		591.4 ± 55.2	657.7 ± 61.8	654.6 ± 55.8		
	∑UFA		2916.3	2625.6	3700.4		
	∑(n-6) PUFA		464.4 ± 46.7	489.8 ± 49.1	541.3 ± 57.8		
	∑(n-3) PUFA		98.5 ± 11.31	90.7 ± 10.32	101.8 ± 3.11		
	∑(n-6)/ ∑ (n-3)		4.7	5.4	5.3		
	∑SFA/∑UFA		0.4	0.8	0.6		

*RT* retention time, Mean ± SE (standard error) of triplicate determinations, *SFA* saturated fatty acids, *MUFA* monounsaturated fatty acids, *PUFA* polyunsaturated fatty acids.

Mean ± SE with different superscript small letters are significantly different from each other (ANOVA followed by Student–Neuman–Keul’s (SNK’s) post-hoc test; *p* < 0.05, n = 3).

A total of 33 fatty acids (FAs) were detected in termite oils from three locations. Saturated fatty acids made up 55% (18 FAs), monounsaturated fatty acids 27% (9 FAs), and polyunsaturated fatty acids 18% (6 FAs). Methyl (9*Z*)-octadecenoate was the most abundant fatty acid, representing over 25% of the total FAs in the termites in each location. The ω-6/ω-3 ratio ranged from 4.7 to 5.3.

**Figure 3:**
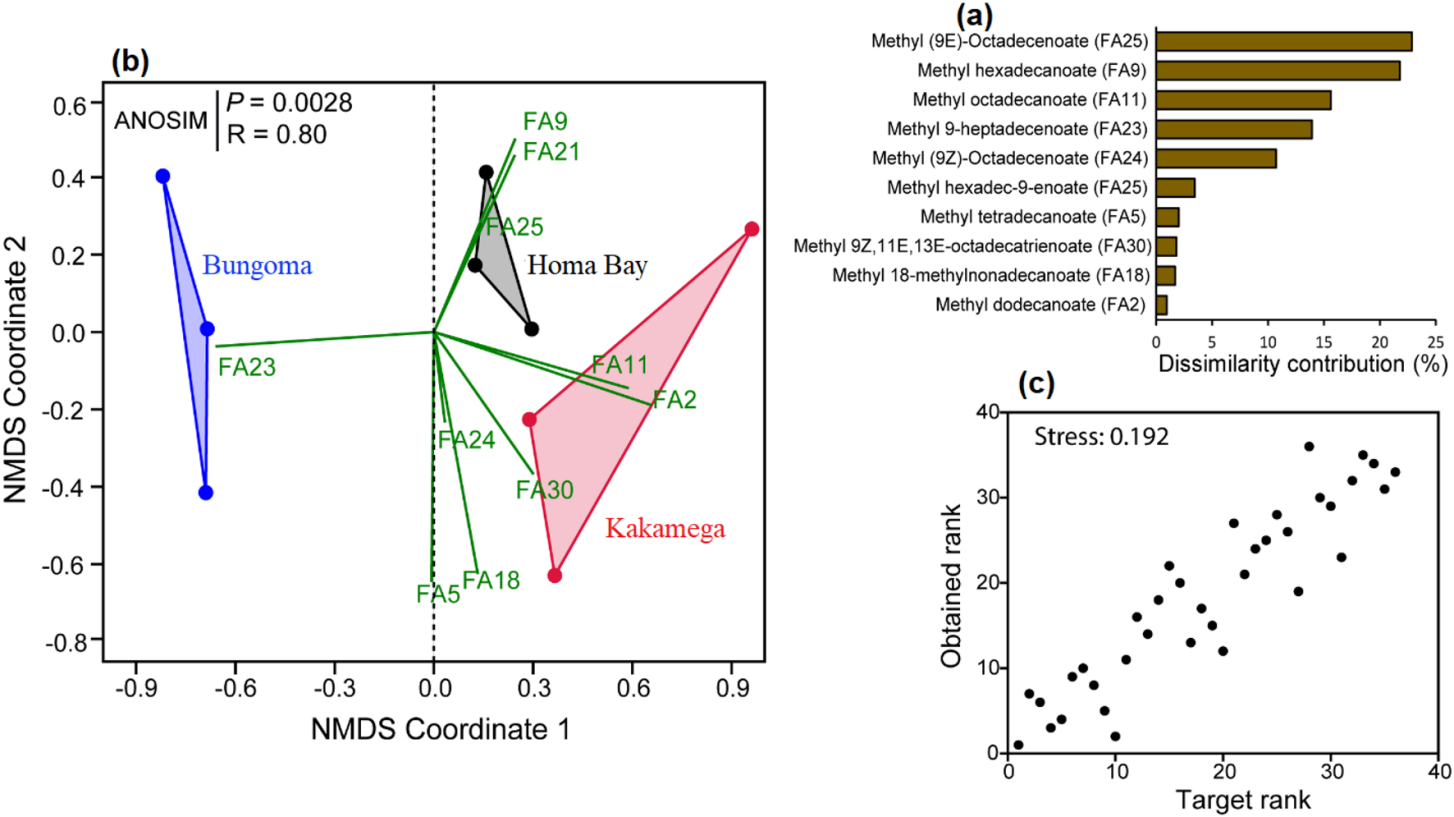
(**a**) Histogram showing the contribution of the 10 most important fatty acids to the discrimination of all the sites. (**b**) Non-metric multidimensional scaling plot (NMDS) clustering the sites based on the fatty acids that were detected in the winged termites oil using the most important fatty acids (**c**) Shepard plot showing the great ordination of the NMDS analysis (stress value < 0.2).

Ten fatty acids were selected as the most important variables for differentiation between the three sites, based on similarity percentage (SIMPER) (Fig. 3a). These fatty acids showed significant variation across the sites (one-way ANOSIM based on Bray–Curtis dissimilarity, R = 0.80, *p* < 0.0028). Using the selected fatty acids, a non-metric multidimensional scaling (NMDS) biplot grouped the sites into three distinct clusters (Fig. 3b) with a weak stress value of 0.192 (great representation of dissimilarities) (Fig. 3c). The results showed that certain fatty acids were associated with specific locations: Bungoma (methyl 9-heptadecenoate), Homa Bay (methyl hexadecanoate, methyl (9*Z*)-hexadecenoate and methyl (9*E*)-hexadecenoate) and Kakamega (methyl dodecanoate, methyl octadecenoate, methyl tetradecanoate, methyl (9Z)-octadecenoate, methyl 18-methylnonadecanoate and methyl (9*Z*,11*E*,13*E*)-octadecatrienoate).

### Amino acids profile

A total of 15 amino acids were detected, including 8 essential and 7 non-essential (Table 3). Significant variations (*p* > 0.05) were observed in the essential amino acids of termites collected from the three sites (Bungoma, Kakamega and Homa Bay). No significant variation was observed in the concentrations of four non-essential amino acids (cystine, glutamic acid, proline and tyrosine). Termites from Homa Bay had higher levels of histidine, isoleucine, leucine, lysine, threonine, and valine. Kakamega termites had higher levels of isoleucine, methionine, and phenylalanine. Termites from Bungoma had relatively lower levels of essential amino acids (except for methionine). Surprisingly, all the samples contained lysine, which is a critical cereal limiting amino acids.

**Table 3:** Amino acids composition (mg/g DM) of termites.

Amino acid	Bungoma	Kakamega	Homa Bay	F_(2,6)_	p-value
Essential amino acids (EAA)					
Histidine	0.9 ± 0.02^a^	1.0 ± 0.00^ab^	1.0 ± 0.03^b^	6.7	0.05
Isoleucine	0.6 ± 0.03^a^	0.7 ± 0.01^b^	0.7 ± 0.00^b^	17.3	0.01
Leucine	0.6 ± 0.04^a^	0.8 ± 0.00^b^	0.9 ± 0.01^c^	46.4	0.001
Lysine	1.0 ± 0.10^a^	1.2 ± 0.00^ab^	1.3 ± 0.03^b^	7.9	0.05
Methionine	0.1 ± 0.00^b^	0.1 ± 0.00^b^	0.08 ± 0.00^a^	9.4	0.05
Phenylalanine	0.3 ± 0.01^a^	0.5 ± 0.00^b^	0.5 ± 0.01^a^	538.6	0.001
Threonine	0.1 ± 0.01^a^	0.1 ± 0.01^a^	0.2 ± 0.00^b^	23.9	0.01
Valine	0.5 ± 0.05^a^	0.6 ± 0.00^ab^	0.7 ± 0.00^b^	8.1	0.05
Non-essential amino acids (NEAA)					
Glycine	0.7 ± 0.06^b^	0.4 ± 0.02^a^	0.4 ± 0.00^a^	12.1	0.01
Alanine	0.1 ± 0.02^ab^	0.1 ± 0.00^a^	0.2 ± 0.00^b^	6.3	0.05
Arginine	0.8 ± 0.23^a^	1.3 ± 0.01^ab^	1.4 ± 0.03^b^	5.9	0.05
Cystine	0.1 ± 0.01^a^	0.1 ± 0.01^a^	0.1 ± 0.00^a^	1.1	ns
Glutamic acid	0.5 ± 0.15^a^	0.3 ± 0.00^a^	0.4 ± 0.02^a^	0.8	ns
Proline	0.7 ± 0.16^a^	0.6 ± 0.01^a^	0.8 ± 0.02^a^	0.8	ns
Tyrosine	0.6 ± 0.10^a^	0.6 ± 0.01^a^	0.7 ± 0.00^a^	1.2	ns

*ns* not significant.

Mean values with different subscripts along each row differ significantly at p > 0.05.

### Mineral composition

Table 4 presents the mineral content of winged termites from the three target sites. Termites from Homa Bay County exhibited significantly higher levels (p < 0.05) of iron, zinc, magnesium, potassium, calcium, and sodium compared to other areas. Likewise, Kakamega termites showed higher concentrations for copper and manganese only. However, lower levels of all the minerals were observed for samples obtained from Bungoma.

**Table 4:** Mineral content (% Dry matter) of winged termites’ powder (mg/100 g).

Minerals	Bungoma	Kakamega	Homa Bay	F_(2,6)_	p-value
Iron	70.7 ± 0.83^a^	105.4 ± 2.37^b^	111.8 ± 0.27^b^	154.2	0.001
Zinc	4.4 ± 0.50^a^	13.9 ± 0.16^b^	16.2 ± 0.11^c^	272.2	0.001
Magnesium	5.0 ± 0.12^a^	10.0 ± 0.10^b^	16.1 ± 0.87^c^	79.9	0.001
Potassium	9.2 ± 0.05^a^	10.9 ± 0.04^b^	12.6 ± 0.09^c^	519.7	0.001
Calcium	33.1 ± 0.08^a^	42.2 ± 0.06^b^	53.0 ± 0.05^c^	14,748.0	0.001
Sodium	14.3 ± 0.08^a^	16.4 ± 0.15^b^	26.4 ± 0.14^c^	1851.5	0.001
Copper	0.3 ± 0.01^a^	1.4 ± 0.02^c^	0.9 ± 0.04^b^	243.8	0.001
Manganese	0.2 ± 0.01^a^	1.0 ± 0.02^c^	0.6 ± 0.03^b^	229.5	0.001

Mean values with different subscripts along each row significantly different at p > 0.05.

### Vitamin composition

The vitamin contents of the winged termites significantly varied p > 0.05 as indicated in Table 5. Copious amounts of vitamins A, C, B12, B1, B2, B3 and B6 were detected in termites from Homa Bay County. However, the lowest levels of all the vitamins were recorded from samples sourced from Bungoma.

**Table 5:** Vitamin content of winged termites’ powder (mg/kg).

Location of termite collection					F_(2,6)_	p-value
Vitamin	Bungoma	Kakamega	Homa Bay			
Vitamin A	2.4 ± 0.40^a^	4.4 ± 0.10^b^	6.4 ± 0.10^c^		67.0	0.001
Vitamin C	0.6 ± 0.08^a^	1.2 ± 0.06^b^	1.9 ± 0.06^c^		90.0	0.001
Vitamin B1	0.3 ± 0.06^a^	0.4 ± 0.06^a^	0.8 ± 0.09^b^		11.6	0.01
Vitamin B2	0.3 ± 0.09^a^	0.4 ± 0.03^a^	0.9 ± 0.02^b^		30.8	0.001
Vitamin B3	0.2 ± 0.06^a^	0.4 ± 0.03^a^	0.9 ± 0.06^b^		54.3	0.001
Vitamin B6	0.4 ± 0.07^a^	0.6 ± 0.03^a^	1.0 ± 0.10^b^		12.2	0.01
Vitamin B9	0.7 ± 0.10^a^	0.9 ± 0.10^a^	1.7 ± 0.40^a^	5.0		ns
Vitamin B12	10.7 ± 0.10^a^	13.5 ± 0.20^b^	17.1 ± 0.10^c^	486.3		0.001

*ns* not significant.

Mean values with different subscripts along each row differ significantly at p > 0.05.

### Proximate composition

There were significant variations (*p* < 0.05) in the proximate constituents of winged termites collected from Bungoma, Kakamega and Homa Bay as illustrated in Table 6. Kakamega termites expressed higher levels of protein whereas Homa Bay termite samples recorded enhanced levels of ash, fibre, fat and carbohydrate contents.

**Table 6:** Proximate composition of winged termites’ powder (% dry matter).

Site	Moisture	Ash	Fibre	Crude protein	Crude fat	Carbohydrates
Bungoma	7.3 ± 0.05^c^	5.5 ± 0.07^b^	5.3 ± 0.03^a^	32.2 ± 0.80^a^	41.2 ± 0.41^a^	0.5 ± 0.02^a^
Kakamega	5.3 ± 0.03^b^	5.2 ± 0.05^a^	6.5 ± 0.12^b^	44.8 ± 0.66^c^	45.5 ± 0.33^b^	6.5 ± 0.14^b^
Homa Bay	4.5 ± 0.01^a^	6.9 ± 0.07^c^	8.2 ± 0.05^c^	37.9 ± 0.54^b^	49.1 ± 0.14^c^	9.4 ± 0.05^c^
F_(2,6)_	1375.9	135.4	255.6	57.8	104.8	1808.1
p-value	0.001	0.001	0.001	0.001	0.001	0.001

Proximate analysis of winged termites is expressed as it is, values with different lowercase letters in a parameter are significantly different from each other.

## Discussion

The discovery of the inhibitive and protective properties of various therapeutic compounds in insects has generated great interest in the scientific community. The exploration of insects for human medicine and nutrition has resulted in the identification of numerous bioactive compounds in species such as edible stink bugs, grasshoppers, *Amphiacusta annulipes*, *Zophobas morio*, *Locusta migratoria*, *Brachytrupes orientalis*, black soldier fly, termites, beetles, desert locusts, caterpillars, and crickets reportedly expressing myriad of bioactive compounds including peptides, isoflavonoids, alkaloids, flavonoids, anthraquinones, tannins, phlobatannins, sterols, triterpenoids and cyanogenic glycosides^19–21^.

Winged termites assessed in this study are no exception, manifesting flavonoids, alkaloids and a cytokinin. Since most of these insects are herbivorous, polyphagous and detritivores, they sequestrate these compounds from plants for subsequent modification into protective metabolites^22^. The identification of the flavonoids, alkaloids and cytokinin from the termites links to their acquisition from the plant materials they feed on.

A study conducted by Ameka et al.^23^ discovered that termites are most attracted to forage on the tree species; Eucalyptus and Grevillea which reportedly exhibit high quantities of flavonoids, alkaloids, tannins, saponins, resins, glycosides and phenols and higher contents of alkaloids, tannins, resins and phenols respectively. Since, the presence of such trees have extensively been documented in Western Kenya^24^, it is plausible that the termites derived the chemicals from them. The notable higher levels of the flavonoids (rutin, quercetin, kaempferol, 4-hydroxybenzoic acid, naringenin), alkaloids (solanine, tomatidine, solanidine, solasodine) and cytokinin (zeatin) in samples from Kakamega may be attributed to the rich diversity of indigenous tropical forest tree species known for high quantities of phytochemicals. The variation in the phytochemical composition of the termites from the different sites may be due to availability and distribution of the specific plant species in those sites and their concentration of such bioactive compounds, as it has been established that dietary sources directly influence the chemical composition of insects^23^. However, the species effect cannot be ruled out in this context since the termites were not specifically identified and therefore Kakamega-based termites may possess genetic adaptations condusive to a heightened sequestration of phytochemicals. This assertion warrants more investigations for scientific clarity.

Flavonoids in foods play a crucial role in human health as antioxidants. The primary sources of flavonoids for humans are fruits and vegetables^25^. These compounds offer a range of benefits, including antioxidant, anti-allergic, anti-inflammatory, anti-diabetic, liver and stomach protective, antiviral, and anti-cancer properties, while alkaloids such as solanine in minute amounts have been found to be anti-cancer and anti-tumor with many others reportedly offering extensive pharmacological benefits^26,27^. Therefore, humans can revamp their therapeutic arsenals against inflammatory infections by consuming winged termites. However, further research is necessary to accurately determine the levels and compositions of flavonoids, phenolics, alkaloids, and other bioactive components in these insects.

The current study confirms that winged termites furnish appreciable levels of oil with samples from Homa Bay and Kakamega manifesting richer sources compared to the samples from Bungoma County. This disparity in oil yields is characteristic of differential environmental conditions notable in terms of dietary sources, temperature and light conditions under which the termites lived^9,28,29^. This oil content plays a critical role in the retention and absorption of flavors contributing to its high palatability, acceptability and reduced applicable oil upon frying and roasting^30^. Additional research using different processing methods and other marketable insect species is needed to confirm these findings.

The dominant fatty acids detected were methyl hexadecanoate, methyl (9*Z*)-octadecenoate (oleic acid) and methyl (9*Z*,12*Z*)-octadecadienoate for SFA, MUFA and PUFA respectively, which corroborates the findings from previous studies^9,30–32^. Also, the concentrations of fatty acids ranged 33–40% SFA, 47–63% MUFA and 11.3–14.3% PUFA which are consistent with values 39.35% SFA, 53.07% MUFA and 7.57% PUFA reported by Fombong and Kinyuru^31^ and underpins the representation in Fig. 2. The fatty acid spectra of the termites collected from the three sites displayed haphazard variation from each other in terms of quantity. This variation may be attributed to differences in developmental stage, sex, biological role, analytical method employed and environmental conditions such as diet and temperature^16,29^. For instance, it has been established that wild collected insects exhibited enhanced levels of both linolenic and linoleic acids compared to commercially reared, grain-fed insects which revealed high linoleic acid but diminished linolenic acid levels^33,34^. Further, certain dynamics in PUFA concentrations were observed when aquatic larval insects feeding on algal diets, metamorphosized into adults, exclusively feeding on terrestrial diets^29^. Insects are metabolically capable of synthesizing SFAs and MUFAs de novo however, they acquire the PUFAs directly from their dietary sources^29^, thus explaining this typical phenomenon.

Termites from the three sites are endowed with PUFAs, particularly, omega 6 methyl (9*Z*,12*Z*)-octadecadienoate, methyl (7*Z*,10*Z*)-hexadecadienoate, methyl (5*Z*,8*Z*,11*Z*,14*Z*)-eicosatetraenoate, omega-3 methyl (9*Z*,12*Z*,15*Z*)-octadecatrienoate (α-Linolenic acid), methyl (5*Z*,8*Z*,11*Z*,14*Z*,17*Z*)-eicosapentaenoate (EPA), omega-5 methyl (9Z,11*E*,13*E*)-octadecatrienoate. The detection of omega-3 fatty acid, EPA, in the present study, that was undetected in previous studies^31,32^, could possibly be reflective of the sensitivities of our extraction and analytical processes used in the various experiments. Omega-3 fatty acids, ALA and EPA have been revealed to avert conditions such as cancer, mental illness, bowel disease, high blood pressure, schizophrenia, cystic fibrosis, and Alzheimer’s disease as well as treatment of skin infections and Crohn’s disease^35,36^. Omega-6 linoleic acids is known to render anticarcinogenic, anti-obesity, antihypertensive and antidiabetic properties to humans^37^. The most abundant MUFA, oleic acid, has been reported to confer anti-cancer and anti-inflammatory properties, and suppression of cardiovascular diseases^38^. The termites also featured a ∑(n-6)/∑(n-3) ratio of 4.7–5.3 which complies with World Health Organization (WHO) recommended daily intake of 5:1^38^. A higher index parallels a significant increase in cardiovascular diseases, inflammatory and chronic diseases. The termites are also noteworthy of UFA/SFA ratios of 0.5–0.9 that agrees with the reported ratio of 0.65^31^ and align with the ratios of 0.43–0.79 in other insects^13^. These ratios imply that termites are sources of highly desirable unsaturated fatty acids, concomitant with high serum high density lipoprotein (HDL) and low risk of atherosclerotic disorders^30^. This unique property has valorized termites as rich food sources in light of their nutritional and pharmacological properties outweighing the negative perception formerly ingrained in the society^11^.

Overall, histidine, lysine and leucine were the most abundant indispensable amino acids, corroborating the observations made by Mabossy-Mobouna et al.^39^ on *Macrotermes bellicosus* while arginine was the predominant dispensable amino acid. Notably, the termites exhibited all the essential amino acids except for tryptophan which has not been detected in previous studies done on related species^39^ thereby portraying termites as complete and balanced rich food source. Histidine, lysine and leucine are critical amino acids for growth^40^ whereas arginine has been reported to enhance calcium absorption^41^. The lower levels of methionine and cysteine certifies the findings of Mabossy-Mobouna et al.^39^ on *M. bellicosus* and further underpins scientific assertions labelling the two sulfur amino acids as the limiting amino acids in most insect species^29^. Additionally, the presence of high lysine qualifies the winged termites for utilization as a complement in the enrichment of lysine-deficient cereal and low protein diets.

Statistical differences were witnessed in the levels of the essential amino acids and certain non-essential amino acids thus endorsing the possible influence of different feed and ecological conditions on amino acids spectra as previously discovered in *Vespa* spp., *Apis* spp. and *Rhynchophorus* spp.^13,42^. Moreover, common variations have also been reported between life stages of completely metamorphosized insect species^43^ possibly due to dissimilarities on the life stages used. However, for majority of insects, the amino acid contents remain fairly constant amidst changes in seasons, geographical areas^42^, and diet or life cycle^29^.

The set of minerals detected in this study mirrors those previously reported in termites^42^. Significant variations of individual minerals in the termites from the three regions may be due to the type of feed, biological role and accumulation of antinutrients in the guts^29^. Specifically, since the termites were collected from different sites, with different ecological and edaphic conditions, the soil types definitely influenced the mineral profiles of the vegetation^44^ which includes the decaying plant matter that termites reportedly feed on^45^, owing to their detritivorous nature in their respective localities. Nonetheless, this study reveals that the termites from Bungoma, Kakamega and Homa Bay bears appreciable amounts of Fe, Zn, Mg, K, Ca, Na, Cu and Mn. Realistically, consumption of 100 g of the termites from the three sites would contribute 3.3–5.3% of 1000 mg Ca Recommended Dietary Intake (RDI), 33.3–155.6% of 0.9 mg Cu RDI, 6.7–33.3% of 3 mg Mn RDI, 321.4–508.2% of 22 mg Fe RDI, 31.4–115.7% of 14 mg Zn RDI and 1.6–5.2% of 310 mg Mg RDI^46^. Our results discern very low Ca levels which has been consistently reported in several insects and explicable by the fact of insects lacking mineralized skeleton^29^. Regardless, Ca plays central role in blood clotting, bone and teeth development^47^.

Trace minerals such as Fe, Zn and Cu are reportedly abundant in termites marked by their potentiality to surpass the RDIs indicated above and reinforcing a tendency demonstrated in other studies^10,16^. Fe is essential in energy metabolism, revamping immunity system, cognitive development, and in physical performance^48^. Mg and Zn prevent cardiomyopathy, muscle degeneration, growth retardation, impaired spermatogenesis, immune-logic dysfunction and bleeding disorder^49^. Mg alone has been affirmed to maintain normal muscles and nerve function, keeps heart rhythm steady, support a healthy immune blood and regulate blood sugar levels^14^. Cu is involved in cellular respiration, peptide amidation, neurotransmitter biosynthesis, pigment formation and strengthening of connective tissue^47^.

Considerable levels of vitamins were detected in winged termites from the three sites. Vitamins are essential dietary components involved in crucial cellular functions but in trace amounts. Except for vitamin A, the levels of the other vitamins in this study were inconsistent with the values reported by other authors^30^. Such differences were also observed in this study where higher concentration of vitamins were depicted in termite samples from Homa Bay County compared to Bungoma and Kakamega Counties. The differences may be claimed to emanate from differences in the substrate type^50^ habitat and preparation methods^51^. Our results discerned higher vitamin B12 which is in agreement with that reported in a study by Oibiokpa et al.^32^. The low levels of the other B vitamins may be attributable to their susceptibility to degradative processing techniques the termites were subjected to^51^. The B vitamins play essential coenzyme roles in the various biochemical and physiological processes^52^. Vitamin A is pivotal to the performance of the visual system, growth and development, and maintenance of epithelial cellular integrity, immune function, and reproduction. On the other hand, vitamin C is essential for growth, development and repair of cells, and involved in a series of body functions such as collagen formation, iron absorption, improved immunity system, wound healing, and the maintenance of cartilage, bones, and teeth^52^. This richness of termites in essential minerals and vitamins justifies their deserved application in combating micronutrient related undernutrition cases^10,49^ by either consuming them in whole forms or incorporating them in other food sources as dietary complement^49^.

The termites from the three sites showed great variability in all the proximate components. The moisture contents ranged between 4.5 and 7.3%, which is slightly lower than that reported by Igwe et al.^30^. This may be attributed to size, maturity and location of collection^53^ and maybe the drying method applied^31^. Moisture content of our products from the various sites are within acceptable storage condition, known to play a pertinent role in suppressing the buildup of microbial loads and reduce chemical deterioration of these products hence influencing their shelf-life^54^. The protein content of Kakamega-sourced termites was remarkably higher compared to that reported by Igwe et al.^30^. The dissimilarities in the protein content of insects have been accredited to differences in the composition of different feeds they forage on, the species, stage of development, sex, climatic condition, and geographical location^48^. The reported protein levels in the current study are of superior quantity to conventional foods of animals and plants origin such as beef, chicken, fish, maize and soya bean^14,47^. Therefore, it can be postulated that consumption of 100 g of the termite products would contribute to 48.8–67.9% of the protein Recommended Daily Allowance (RDA) of 0.66 g/kg adult/day^55^ that is in agreement with the reported edible insects’ contribution to the RDA of 23–56%^53^. The protein content of termites has been reported to be of high quality and digestibility, therefore proved ideal for alleviation and management of food malnutrition cases^11,15^.

The noticeable variability of fat contents may be ascribed to differences in sex, season of collection, stage of development, habitat and feed either individually or in combination^56,57^. The fat was the predominant component of the termites in this study which confirms previous reports^31^. The fat acts as the main energy source, provide fatty acids profiles, harbors fat soluble compounds like fat soluble vitamins^56^ and volatiles^38^. The ash contents of the termites slightly differed from the reported values of 6.2–7.2%^15^ and 7.6%^30^. Ash is known to represent the minerals in a food and actively promote metabolism of organic compounds such as carbohydrates and fats^14^. Fibre contents of termites has been purported to be chiefly chitin which comprise approximately10% of whole insects^31^.

In this study, significant differences in the fibre contents were apparent with Homa Bay termite samples exhibiting the highest value. The values were however comparable to 6.2–7.2% in termites^58^. Dietary fibre is lauded for promoting peristaltic movement in the gut during digestion as well as imparting a sense of satiety that translates to reduced fat and energy intake which is ideal for weight control^53^. The chitin also serves as prebiotic known to enhance the establishment of beneficial probiotic bacteria that consequently improve gut health, confers anti-inflammatory^3^, antimicrobial, antitumor and antioxidant properties^57^. Great significant variations in carbohydrates were evident in this study. Generally, it has been revealed that insects are poor sources of carbohydrate, possibly inadequate to even satisfy the adult human requirement of 400–500 g^53^. For this reason, they have been regarded as low carbohydrate-high protein food hence recommended exemplary fit diet for control of cardiovascular diseases and weight gain^48^. Overall, the chemical integrants in the present study manifested remarkable variabilities among the termites from the three sites. Notably, the specific identification of the experimental termites was not undertaken, raising the possibility that these variabilities could stem from species disparities and potential contamination or adulteration, given the known diversity of termite species across different geographical locations^15^. Additionally, development of toolkits for rapid species identification in edible insects should be considered. Therefore, this remains the study limitation that future outlooks of similar nature should take into consideration.

## Conclusion

This study sheds light on the nutritional resourcefulness of dried and marketed termite swarmers or “alates” [i.e., winged termites] in Western Kenya. The identification of various flavonoins, alkaloids, and a cytokinin in termites expands our understanding of the diverse array of phytochemicals present in these insects, thereby providing scientific validation for their historical use in local diets and therapeutic practices. This finding offers a strong foundation for further exploration of termites from a pharmacological perspective, emphasizing the importance of extending phytochemical assessments to termites of the same species across different geographical regions to elucidate influence of location on chemical composition. Furthermore, our study highlights termites as rich sources of extractable oils, abundant in monounsaturated and polyunsaturated fatty acids, notably omega-3 and omega-6, which hold promising applications in both culinary and cosmetic industries. Additionally, the high protein content and appreciable levels of essential amino acids (such as histidine, lysine, and leucine) and minerals (including zinc, iron, and calcium) present in termites underscore their potential in formulating modern foods, particularly of plant origin, to address malnutrition in the region. However, the exploitation of termites for food and other resources may be impeded by seasonal availability and their rearing may prove difficult owing to their complexed social structures. Further research directed towards rearing termites on feed-specific diets which guarantees maximum derivable chemicals and nutrients, for mass production of nutrient and phytochemical rich termites is necessary. Also, research involving comparison of the chemical composition of different edible life stages of termites consumed by other communities in Africa should be considered.

## Methods

### Insect obtainment and processing

Representative samples of sun-dried winged termite (*Macrotermes* spp.) (alates) were purchased from three local markets in Western Kenya namely Bungoma (0.5695° N, 34.5584° E), Kakamega (0.2827° N, 34.7519° E) and Homa Bay (0.6221° S, 34.3310° E). The samples were purchased from ten vendors in each town/market during the swarming period of the termites. From each vendor, termite samples sun-dried within 24 h, collectively weighing 2 kg were obtained. The samples were then stuffed into polyethylene sterile zip lock bags, appropriately labeled and ice-packed in cooler boxes. The samples were then transported to International Center of Insect Physiology and Ecology (*icipe*) laboratory, Nairobi, Kenya within 24 h after collection and immediately stored at − 80 °C awaiting further processing and analysis.

### Phytochemicals analysis

Flavonoids, alkaloids and cytokinin levels in the termite samples were determined according to procedures described by Matsuura and Fett-Neto^27^ with slight modifications. Termites ground into powder in liquid nitrogen (1 g) were mixed with 10 mL of 80% methanol (v/v) and vortex-shaken for 1 min and ultra-sonicated for 1 h to enhance phytochemicals extraction. The remaining residues were further re-extracted twice and later pooled together. The extracts were then centrifuged at 4200 rpm for 15 min, the supernatant filtered through a Whatman filter paper No. 32 and the filtrate analyzed by LC–MS. The chromatographic separation was achieved on Agilent 1260 Infinity HPLC system (Agilent Technologies, Palo Alto, CA) coupled to an Agilent 6120 mass detector MS with a single quadrupole analyzer (Agilent Technologies, Palo Alto, CA) using ZORBAX SB-C18, 4.6 × 250 mm, 3.5 μm column, operated at 40 °C. Mobile phases used were made up water (A) and acetonitrile (B) each with 0.01% formic acid. The following gradient system was used: (0.01 min, 5% B; 0.01–5 min, 5% B; 5–10 min, 5–20% B; 10–15 min, 20% B; 15–20 min, 20–80% B; 20–25 min, 80% B; 25–30 min, 80–100% B; 30–35 min, 100% B; 35–37 min, 100–5% B; 37–42 min, 5% B. The flow rate was held constant at 0.5 mL/min and the injection volume was 3 μL. The LC was interfaced to a quadruple mass spectrometer operated on ESI positive mode at a mass scan range of m/z 100–2000. The dwell time for each ion was 50 ms. Other parameters of the mass spectrometer were as follows: capillary voltage, 3.0 kV; cone voltage, 30 V; extract voltage, 5 V; RF voltage, 0.5 V; source temperature, 110 °C; nitrogen gas temperature for desolvation, 380 °C; and nitrogen gas flow for desolvation, 400 L/h. Authentic standard of rutin hydrate (≥ 94%, Sigma–Aldrich (St Louis, MO) was serially diluted (1–100 ng/μL) and also analyzed by the LC–MS to yield linear calibration curves (peak area vs. concentration) with equation: [y = 5578.4x − 39,094 (R^2^ = 0.9990)] was used for external quantification of flavonoids, alkaloids and cytokinin. The identities of flavonoids, alkaloids and cytokine were confirmed with commercially-purchased samples by co-injections. Three replicates were carried out with each replicate done on a different termite’s samples.

### Extraction of winged termite oil

Ground insect samples (50 g) were separately mixed with 250 mL solvent solution (dichloromethane—methanol (2:1 v/v) containing butylated hydroxyl toluene (10 mg/L) to extract the oil, as previously described^59^ with some modifications. The mixture was manually shaken for 30 s, sonicated for 1 h, followed by centrifugation (1500 g, 23 °C, 5 min). The supernatant was then collected into a separating funnel mixed with 100 mL of 0.9% NaCl solution, shaken vigorously and allowed to stand until the biphasic system appeared. The upper aqueous phase was discarded. The lower phase was passed through a plug containing anhydrous sodium sulfate into a pre-weighed 500 mL rotary flasks and the solvent removed *in vacuo* before storing the oil sample at − 20 °C for further analysis. The following equation was used to compute the percentage (%) of the extracted oils (yield):

$$\frac{\left[\left(weight \,of\, rotary\, flask+oil\right)-\left(the \,weight\, of\, the \,rotary\,flask\right)\right]}{(initial\, weight \,of \,the \,sample) }\times 100.$$

### Determination of fatty acids profile

The fatty acid (FA) compositions of the three termites’ oil (100 mg), were converted to the fatty acid methyl esters by adding 1 mL sodium methoxide in dry methanol (15 mg/mL) at 60 °C for 1 h. Thereafter, the reaction was quenched by adding 100 μL of deionized water followed by a 1 min vortexing. The resulting methyl esters were extracted using gas chromatography (GC)-grade hexane (1 mL; Sigma–Aldrich, St. Louis, MO, USA) and centrifuged at 14,000 rpm for 5 min. The supernatant was dried over anhydrous Na_2_SO_4_ and analyzed (1.0 μL) by GC–MS (7890A gas chromatograph; Agilent Technologies, Inc., Santa Clara, CA, USA) coupled to a 5975 C mass selective detector (Agilent Technologies, Inc., Santa Clara, CA, USA). The GC was fitted with a (5%-phenyl)-methylpolysiloxane (HP5 MS) low bleed capillary column of (30 m × 0.25 mm i.d., 0.25 µm; J&W, Folsom, CA, USA). Helium was the carrier gas, and the column flow rate was 1.25 mL/min. The GC’s inlet temperature was maintained at 270 °C, while that of the transfer line was set at 280 °C. The column oven’s temperature was preset to rise from 35 to 285 °C, with the former temperature being held for 5 min followed by a 10 °C increase for every minute until the temperature reached 280 °C, where it was maintained for 20.4 min. The mass selective detector was maintained at quadrupole (180 °C) and ion source (230 °C) temperatures. Electron impact (EI) mass spectra was obtained at the acceleration energy of 70 eV with fragment ions being analyzed over 40–550 m/z mass range in the full scan mode, having a solvent delay time set at 3.3 min. The extracting solvent (hexane), blank machine runs was similarly analyzed and their peaks excluded from analysis. Fatty acids were identified via their methyl esters using comparisons of their fragmentation patterns and retention times to those of known fatty acid methyl ester standards where available and their reference spectra published by library–MS databases: National Institute of Standards and Technology (NIST) 05, 08, and 11. Authentic sample of methyl octadecenoate (0.2–125 ng/μL) was subsequently analyzed under the same GC–MS conditions to obtain a linear calibration curve [y = 7E + 06x − 4E + 07; (R^2^ = 0.9757)] that was used for external quantification of the fatty acid methyl esters. The data are expressed as µg/mg of detected total analyzed fatty acids.

### Amino acids determination

The amino acid composition was determined as previously described^60^. Samples (100 mg) were hydrolyzed with 1.5 mL of 6N HCl at 110 ℃ for 24 h under nitrogen to completion. The hydrolysates were vacuum-evaporated to dryness at 40 °C and subsequently reconstituted in 1 mL of 0.01% formic acid-acetonitrile (95:5) mix, vortexed for 30 s, sonicated for 30 min, and then centrifuged at 14,000 rpm for 15 min. The supernatant was filtered using 0.20 μm membrane filter (Life Science, USA) and analyzed (0.2 μL) by LC–MS with conditions similar to the phytochemicals analysis section with exception of mass scan range of m/z 50–600 and gradient system (0–8 min, 10% B; 8–14 min,10–100% B; 14–19 min, 100% B; 19–21 min, 100–10% B; 21–25 min, 10% B). Mass spectrometric data, retention time, and co-injection of the hydrolysate with an authentic standard amino acid mixture were used to identify the amino acids. Amino acid standard solution (AAS 18) obtained from Sigma-Aldrich (1–105 µg/µL, Chemie GmbH, Munich, Germany) was similarly analyzed by LC–MS and used for external quantification of the amounts of each amino acid present. This was repeated three times using different batch of termite’s samples.

### Mineral analysis

The winged termite powder (1 g each) was incinerated to ash in a muffle furnace at 550 ℃ overnight. The resulting ashes were digested in 6N HCl and the content of the various minerals determined using atomic absorption spectrometry (Shimadzu, AA-6300, Tokyo, Japan) according to previously published methods^61^.

### Vitamins determination

#### Water soluble vitamins

Water soluble vitamins determinations followed the procedures delineated by Thermo Fisher Scientific^62^. The termite samples (100 mg) were mixed with 25 mL of distilled water, sonicated for 15 min and filtered through 0.20 μm membrane filter (Life Science, USA) into UPLC vials. The chromatographic analysis was performed on a Liquid chromatographic system with Diode Array Detector (LC-30AC with Nexera column oven CTO-30A, Shimadzu, Tokyo, Japan) equipped with a Phenomenex C18 Column Synergi 100 × 3.00 mm, 2.6 µm polar (Phenomenex, Torrance, CA, USA) at 30 °C. A gradient elution consisting of two solvents; A: 25 mM phosphate buffer and B: 7:3 v/v Acetonitrile-Mobile phase A constituted the mobile phase. Chromatographic separations were set to run for 12 min with a flow rate of 0.4 mL/min. Stock solutions (1.0 mg/mL), prepared by dissolving the individual water-soluble vitamin standards in distilled water except for Vitamin B_2_ in (5 mM potassium hydroxide) and Vitamin B_9_ in (20 mM potassium hydrogen carbonate) was used to make four standards of concentrations 2, 5, 10 and 15 µg/mL to generate a calibration curve (R^2^ = 0.996) for external quantification of the respective vitamins. All determinations were carried out in triplicates.

#### Fat-soluble vitamins

Fat-soluble vitamins were assessed in consonance with methods outlined by Bhatnagar-Panwar et al.^63^. Samples (500 mg) were homogenized with 6 mL of ethanol premixed with 0.1% BHT. Potassium hydroxide (80% w/v) (120 µL) was then added to the resultant homogenate, vortexed for 1 min, incubated at 80 °C for 5 min and subsequently ice-cooled. The mixture was then mixed with 4 mL of deionized water and vortexed for 1 min. For extraction, 5 mL HPLC-grade hexane (Sigma–Aldrich, St. Louis, MO, USA) (5 mL) was added to the mixture followed by a-5 min centrifugation at 3000 rpm to extract the resulting fat-soluble vitamins. The supernatants were extracted and pellets re-extracted twice using hexane into separate test tubes and the upper phases subsequently pooled. The extracts were dried under nitrogen and the residue reconstituted in 1 mL of methanol: tetrahydrofuran (85:15 v/v), vortexed for 1 min, sonicated for 30 s and filtered into HPLC sample vials. Sample analysis (10 μL) with a total flow rate of 0.4 mL/min was performed using reverse-phase HPLC (Shimadzu Nexera UPLC system) linked to SPD-M2A detector. The UPLC was equipped with a YMC C30, carotenoid column (3 µm, 150 × 3.0 mm, YMC Wilmington, NC). A gradient elution consisting of two solvents; A: methanol/tert-butyl methyl ether/water (85:12:3, v/v/v, with 1.5% ammonium acetate in the water) and B: methanol/tert-butyl methyl ether/water (8:90:2, v/v/v, with 1% ammonium acetate in the water). Compounds presenting the eluting sample were monitored at 290 nm. Peaks were identified by their retention time and absorption spectra were compared to those of known standards (Sigma Chemicals). All determinations were done in three replicates.

### Proximate analysis of winged termites

Proximate analysis (Dry mater, crude protein, and total ash) was carried out based on official methods of Association of Official Analytical Chemists^64^. The moisture content was determined by drying the ground winged termites in an oven at 105 °C for 24 h. The nitrogen content was determined using an automatic Kjeldahl analyzer (Velp, Scientifica, Europe) after digestion in a concentrated H_2_SO_4(l)_ the Kjeldahl method and later converted to crude protein content using a nitrogen-to-protein conversion factor 6.25. Crude ash was done gravimetrically in a muffle furnace at 550 °C for 3 h. Crude fibre was determined by acid digestion and loss on ignition in a fiber analyzer (Velp Scientifica, Europe). The crude fat content was determined by diethyl ether extraction in a fat extraction unit (Velp Scientific, Usmate, Italy) following the Randall technique. Total carbohydrate was calculated by difference using standard methods^64^. All parameters discussed above were determined in quadruplicate per sample site and expressed as a percentage.

### Statistical analyses

Data from oil yield were normally distributed (Shapiro–Wilk test: *p* > 0.05) and their variance were similar (Bartlett’s test: p > 0.05), therefore, we used the unpaired *F*-test to compare the oil yield between the three sites. To visualize the relative abundance of the three classes of fatty acids (saturated fatty acid (SFA), monounsaturated fatty acid (MUFA), and polyunsaturated fatty acid (PUFA)) in termites from the three study sites, we generated 100% stacked bars using excel and compared their proportions using one-way ANOVA. We employed the test of analysis of variance (ANOVA) followed by the Student–Neuman–Keuls (SNK) post-hoc multiple comparisons test to compare the concentration of individual fatty acids across the various sites. To analyze the fatty acids profiles of different termites from the three experimental sites, one-way analysis of similarities (ANOSIM) utilizing the Bray–Curtis dissimilarity matrix was used. The relative contribution of different fatty acids to the dissimilarity of termites from the different sites was calculated and visualized using the non-metric multidimensional scaling approach based on the similarity percentages (SIMPER) analysis. Analysis of variance and Tukey’s multiple comparison option were employed to establish the significance difference (α = 0.05) in the proximate, amino acids, minerals, vitamins and phytochemical contents of the termites from the three sites, with the aid of ‘car’ package and ‘agricolae’ package in R Studio software version 2022-07-22 for Windows^65^. Unless specified otherwise, all measurements were conducted in triplicate and data presented as means ± SE.

## Data Availability

The data that support the findings of this study are available from the corresponding author upon reasonable request.

## References

[1] Adeyanju D (2023). Assessing food security among young farmers in Africa: Evidence from Kenya, Nigeria, and Uganda. Agric. Food Econ..

[2] Adeyeye SAO, Ashaolu TJ, Bolaji OT, Abegunde TA, Omoyajowo AO (2023). Africa and the Nexus of poverty, malnutrition and diseases. Crit. Rev. Food Sci. Nutr..

[3] Stull VJ (2021). Impacts of insect consumption on human health. J. Insects Food Feed..

[4] KNBS. *Kenya Demographic and Health Survey 2014*. 10.4324/9780203798935-17. (2015).

[5] Nadeau L, Nadeau I, Franklin F, Dunkel F (2015). The potential for entomophagy to address undernutrition. Ecol. Food Nutr..

[6] Van Huis A (2013). Edible Insects. Future Prospects for Food and Feed Security. Food and Agriculture Organization of the United Nations.

[7] Van Huis A (2016). Edible insects are the future?. Proc. Nutr. Soc..

[8] Kelemu S (2015). African edible insects for food and feed: Inventory, diversity, commonalities and contribution to food security. J. Insects Food Feed..

[9] Kinyuru JN (2021). Oil characteristics and influence of heat processing on fatty acid profile of wild harvested termite (*Macrotermes subhylanus*) and long-horned grasshopper (*Ruspolia differens*). Int. J. Trop. Insect Sci..

[10] Hlongwane ZT, Slotow R, Munyai TC (2020). Nutritional composition of edible insects consumed in Africa: A systematic review. Nutrients.

[11] de Figueirêdo RECR, Vasconcellos A, Policarpo IS, Alves RRN (2005). Edible and medicinal termites: A global overview. J. Ethnobiol..

[12] Roos N, Van Huis A (2017). Consuming insects: Are there health benefits?. J. Insects Food Feed..

[13] Rumpold BA, Schlüter OK (2013). Nutritional composition and safety aspects of edible insects. Mol. Nutr. Food Res..

[14] Ojinnaka MC, Ofoelo MU, Ezenwa LI (2015). Nutritional evaluation of wheat cakes enriched with edible African termites (*Macrotermes nigeriensis*). Agro-Science.

[15] Kinyuru JN (2013). Nutrient composition of four species of winged termites consumed in western kenya. J. Food Compos. Anal..

[16] Akullo J, Agea JG, Obaa BB, Okwee-Acai J, Nakimbugwe D (2018). Nutrient composition of commonly consumed edible insects in the Lango sub-region of northern Uganda. Int. Food Res. J..

[17] Kinyuru JN (2012). Identification of traditional foods with public health potential for complementary feeding in western Kenya. J. Food Res..

[18] Abu T, Njoku-Onu KA, Augustine EU (2017). Classification, chemical composition and therapeutic properties of termite species—A review. Int. J. Community.

[19] Ssepuuya G (2021). Suitable extraction conditions for determination of total anti-oxidant capacity and phenolic compounds in *Ruspolia differens* Serville. J. Insects Food Feed..

[20] Ochieng BO (2022). Dynamics in nutrients, sterols and total flavonoid content during processing of the edible long-horned grasshopper (*Ruspolia differens* Serville) for food. Food Chem..

[21] Cheseto X (2015). Potential of the desert locust *Schistocerca gregaria* (Orthoptera: Acrididae) as an unconventional source of dietary and therapeutic sterols. PLoS ONE.

[22] Musundire R, Zvidzai CJ, Chidewe C, Samende BK, Manditsera FA (2014). Nutrient and anti-nutrient composition of *Henicus whellani* (Orthoptera: Stenopelmatidae), an edible ground cricket, in south-eastern Zimbabwe. Int. J. Trop. Insect Sci..

[23] Ameka CM, Muok B, Oyieke H (2021). Analysis of feed preference of edible termites (Isoptera) on selected plants and their crude extract phytochemistry. Adv. Entomol..

[24] Otieno NE, Analo C (2012). Local indigenous knowledge about some medicinal plants in and around Kakamega forest in western Kenya. F1000Research.

[25] Kaur C, Kapoor HC (2008). Antioxidants in fruits and vegetables—The millennium’s health. Int. J. Food Sci. Technol..

[26] Musundire R, Osuga IM, Cheseto X, Irungu J, Torto B (2016). Aflatoxin contamination detected in nutrient and anti-oxidant rich edible stink bug stored in recycled grain containers. PLoS ONE.

[27] Matsuura HN, Fett-Neto AG, Gopalakrishnakone P, Carlini CR, Ligabue-Braun R (2015). Plant alkaloids: Main features, toxicity, and mechanisms of action. Plant Toxins.

[28] Tzompa Sosa DA, Fogliano V, Shields VD (2017). Potential of insect-derived ingredients for food applications. Insect Physiology and Ecology.

[29] Oonincx DGAB, Finke MD (2020). Nutritional value of insects and ways to manipulate their composition. J. Insects Food Feed..

[30] Igwe CU, Ujowundu CO, Nwaogu LA, Okwu GN (2011). Chemical analysis of an edible African termite, *Macrotermes nigeriensis*; a potential antidote to food security problem. Biochem. Anal. Biochem..

[31] Fombong F, Kinyuru J, Khan MA, Ahmad W (2018). Termites as food in Africa. Termites and Sustainable Management.

[32] Oibiokpa FI, Akanya HO, Jigam AA, Saidu AN (2017). Nutrient and antinutrient compositions of some edible insect species in northern Nigeria. FUJNAS.

[33] Sushchik NN (2013). Comparison of fatty acid contents and composition in major lipid classes of larvae and adults of mosquitoes (Diptera: Culicidae) from a steppe region. Insect Sci..

[34] Fontaneto D (2011). Differences in fatty acid composition between aquatic and terrestrial insects used as food in human nutrition. Ecol. Food Nutr..

[35] Glick NR, Fischer MH (2013). The role of essential fatty acids in human health. J. Evid. Based Complement. Altern. Med..

[36] Kaur N, Chugh V, Gupta AK (2014). Essential fatty acids as functional components of foods—A review. J. Food Sci. Technol..

[37] Koba K, Yanagita T (2013). Health benefits of conjugated linoleic acid (CLA). Obes. Res. Clin. Pract..

[38] Cheseto X, Baleba SBS, Tanga CM, Kelemu S, Torto B (2020). Chemistry and sensory characterization of a bakery product prepared with oils from African edible insects. Foods.

[39] Mabossy-Mobouna G, Latham P, Malaisse F (2020). Chemical aspects of human consumption of termites in Africa. Geo. Ecol. Trop..

[40] Parikh P (2022). Animal source foods, rich in essential amino acids, are important for linear growth and development of young children in low-and middle-income countries. Matern. Child Nutr..

[41] Ademola OA, Omolara AH, Abioye OR (2017). Amino acids profile of bee brood, soldier termite, snout beetle larva, silkworm larva, and pupa: Nutritional implications. Adv. Anal. Chem..

[42] Meyer-Rochow VB, Gahukar RT, Ghosh S, Jung C (2021). Chemical composition, nutrient quality and acceptability of edible insects are affected by species, developmental stage, gender, diet, and processing method. Foods.

[43] Pieterse E, Pretorius Q (2013). Nutritional evaluation of dried larvae and pupae meal of the housefly (*Musca domestica*) using chemical-and broiler-based biological assays. Anim. Prod. Sci..

[44] Joy EJM (2015). Soil type influences crop mineral composition in Malawi. Sci. Total Environ..

[45] Okullo P, Greve PMK, Moe SR (2013). Termites, large herbivores, and herbaceous plant dominance structure small mammal communities in savannahs. Ecosystem.

[46] Alimentarius C (2019). Codex Nutrient Reference Values.

[47] Ghosh S, Lee S-M, Jung C, Meyer-Rochow VB (2017). Nutritional composition of five commercial edible insects in South Korea. J. Asia Pac. Entomol..

[48] Kuntadi K, Adalina Y, Maharani KE (2018). Nutritional compositions of six edible insects in Java. Indones. J. For. Res..

[49] Alamu OT, Amao AO, Nwokedi CI, Oke OA, Lawa IO (2013). Diversity and nutritional status of edible insects in Nigeria: A review. Int. J. Biodivers. Conserv..

[50] Tang C (2019). Edible insects as a food source: A review. Food Prod..

[51] Mlček J, Rop O, Borkovcova M, Bednářová M (2014). A comprehensive look at the possibilities of edible insects as food in Europe—A review. Pol. J. Food Nutr. Sci..

[52] FAO (2002). Human Vitamin and Mineral Requirements.

[53] Ekop EA, Udoh AI, Akpan PE (2010). Proximate and anti-nutrient composition of four edible insects in Akwa Ibom state, Nigeria. World J. Appl. Sci. Technol..

[54] Hernández-Álvarez A-J (2021). Drying technologies for edible insects and their derived ingredients. Dry. Technol..

[55] WHO, FAO, UNU (2007). Protein and Amino Acid Requirements in Human Nutrition.

[56] Adámková A (2017). Nutritional potential of selected insect species reared on the island of Sumatra. Int. J. Environ. Res. Public Health.

[57] Ojha S, Bekhit AE, Grune T, Schlu OK (2021). Bioavailability of nutrients from edible insects. Curr. Opin. Food Sci..

[58] Gahukar RT (2011). Entomophagy and human food security. Int. J. Trop. Insect Sci..

[59] Igiehon NO, Babalola OO, Cheseto X, Torto B (2021). Effects of rhizobia and arbuscular mycorrhizal fungi on yield, size distribution and fatty acid of soybean seeds grown under drought stress. Microbiol. Res..

[60] Cheseto X (2018). Identification of glutamic acid as a host marking pheromone of the African fruit fly species *Ceratitis rosa* (Diptera: Tephritidae). J. Agric. Food Chem..

[61] Poitevin E (2016). Official methods for the determination of minerals and trace elements in infant formula and milk products: A review. J. AOAC Int..

[62] Scientific, T. F. *Determination of Water- and Fat-Soluble Vitamins by HPLC*. *Knowledge Creation Diffusion Utilization.* http://www.dionex.com/en-us/webdocs/88784-TN89-HPLC-WaterFatSolubleVitamins-27Oct2010-LPN2598.pdf (2017).

[63] Bhatnagar-Panwar M, Bhatnagar-Mathur P, Bhaaskarla VV, Dumbala SR, Sharma KK (2015). Rapid, accurate and routine HPLC method for large-scale screening of pro-vitamin A carotenoids in oilseeds. J. Plant Biochem. Biotechnol..

[64] AOAC (1990). Official Methods of Analysis of the AOAC.

[65] R Core Team & R Development Core Team. *A Language and Environment for Statistical Computing*. https://www.r-project.org (R Foundation for Statistical Computing, 2018).

